# The Bright Sides of Sadness in Late Life

**DOI:** 10.1093/geroni/igaa057.2139

**Published:** 2020-12-16

**Authors:** Claudia Haase, Deborah Wu, Sandy Lwi, Alice Verstaen, Robert Levenson

**Affiliations:** 1 Northwestern University, Evanston, Illinois, United States; 2 University of Massachusetts Amherst, Amherst, Massachusetts, United States; 3 University of California, Berkeley, Berkeley, California, United States

## Abstract

Sadness is often thought of as unpleasant and dysfunctional. Yet, evolutionary-functionalist approaches and discrete emotional aging frameworks suggest that sadness is an emotion that helps us deal with loss and thus may become particularly salient and adaptive in late life. This talk presents findings from a multi-study, multi-method research program using age-diverse samples and experimental and longitudinal study designs. Findings show (1) intact or elevated levels of sadness responding in late life (i.e., higher sadness expressions in response to distressing film clips; higher coherence between sad facial expressions and autonomic physiology in response to film clips depicting loss; stability in sadness behaviors in marital conflict interactions). Moreover, (2) higher levels of sadness responding are linked to adaptive outcomes in late life (i.e., higher social connectedness, higher compensatory control strategies) with some effects generalizing across age groups (i.e., links between sadness coherence and well-being). Implications for future research are discussed.

